# The Development of a Genome Wide SNP Set for the Barnacle Goose *Branta leucopsis*

**DOI:** 10.1371/journal.pone.0038412

**Published:** 2012-07-10

**Authors:** Rudy M. Jonker, Qiong Zhang, Pim Van Hooft, Maarten J. J. E. Loonen, Henk P. Van der Jeugd, Richard P. M. A. Crooijmans, Martien A. M. Groenen, Herbert H. T. Prins, Robert H. S. Kraus

**Affiliations:** 1 Resource Ecology Group, Wageningen University, Wageningen, the Netherlands; 2 Department of Animal Behaviour, University of Bielefeld, Bielefeld, Germany; 3 Key Laboratory of Animal Ecology and Conservation Biology, Institute of Zoology, Chinese Academy of Sciences, Beijing, China; 4 Arctic Centre, University of Groningen, Groningen, the Netherlands; 5 Dutch Centre for Avian Migration & Demography, Netherlands Institute of Ecology, Wageningen, the Netherlands; 6 Animal Breeding and Genomics Centre, Wageningen University, Wageningen, the Netherlands; 7 Conservation Genetics Group, Senckenberg Research Institute and Natural History Museum, Gelnhausen, Germany; University of Uppsala, Sweden

## Abstract

Migratory birds are of particular interest for population genetics because of the high connectivity between habitats and populations. A high degree of connectivity requires using many genetic markers to achieve the required statistical power, and a genome wide SNP set can fit this purpose. Here we present the development of a genome wide SNP set for the Barnacle Goose *Branta leucopsis*, a model species for the study of bird migration. We used the genome of a different waterfowl species, Mallard *Anas platyrhynchos*, as a reference to align Barnacle Goose second generation sequence reads from an RRL library and detected 2188 SNPs genome wide. Furthermore, we used *chimeric* flanking sequences, merged from both Mallard and Barnacle Goose DNA sequence information, to create primers for validation by genotyping. Validation with a 384 SNP genotyping set resulted in 374 (97%) successfully typed SNPs in the assay, of which 358 (96%) were polymorphic. Additionally, we validated our SNPs on relatively old (30 years) museum samples, which resulted in a success rate of at least 80%. This shows that museum samples could be used in standard SNP genotyping assays. Our study also shows that the genome of a related species can be used as reference to detect genome wide SNPs in birds, because genomes of birds are highly conserved. This is illustrated by the use of *chimeric* flanking sequences, which showed that the incorporation of flanking nucleotides from Mallard into Barnacle Goose sequences lead to equal genotyping performance when compared to flanking sequences solely composed of Barnacle Goose sequence.

## Introduction

Migration of animals is one of the most visible natural phenomena and as such has attracted much scientific attention. Because migrants connect habitats, migratory species can play a key role in understanding how local environmental changes affect populations and habitats at a larger scale [1]. Additionally, migratory birds, especially waterfowl such as geese and ducks, are thought to play an important role in the spread of infectious diseases such as Avian Influenza [2], [3]. More insight into the genetic population structure of migratory species will be helpful in understanding migration patterns and possible migration changes [4]. Previous genetic studies on geese used microsatellites with varying success. For example, Anderholm *et al.* [5] successfully showed nest parasitism in barnacle geese using 14 microsatellites, while Harrison *et al.* [6], using 15 microsatellite markers, could not discover population structure among 1127 light-bellied brent geese *Branta bernicla hrota*. However, because of the high connectivity between migratory populations high discriminating power is needed to disentangle population structure, especially when insight in recent migratory changes is desired. The detection and development of Single Nucleotide Polymorphisms (SNPs) could fill this knowledge gap for migratory species since the statistical power of SNPs, of which hundreds can nowadays be easily applied in a single study, is considerably higher than of microsatellites [7], [8]. To our knowledge, for migratory birds only for the Mallard (*Anas platyrhynchos*), which is a partial migrant, SNPs have been described genome wide [9]. The Barnacle Goose is one of the model species for migration research, studied especially for its flexibility in adjusting migration schedules to ecological changes [10]–[15]. The Barnacle Goose has three different flyways [16], which are assumed to have little exchange [17]. Within the Russian flyway there are several populations, of which the Swedish and Dutch were established recently [10], [18], [19]. The development of large SNP sets makes it possible to analyse demography and recent development of new populations. Due to migratory changes problems occur such as increasing crop damage resulting in societal debate on whether conservation of geese is still needed or how crop damage can be reduced. Moreover, geese are important poultry species such as several varieties of Greylag Goose *Anser anser*. Although barnacle geese are not used in agricultural production, the detection of SNPs in Barnacle Goose may provide potential SNPs for related species and their domesticated forms.

Kerstens *et al*. (2009) [20] and Van Bers *et al* (2010) [21] showed the efficient use of next generation sequencing for the detection of a large amount of SNPs without having a sequenced reference genome (in Turkey *Meleagris gallopavo* and Great Tit *Parus major* respectively). These studies created an incomplete genome from short sequences stemming from next generation Illumina sequencing and used that as a reference genome for SNP detection. The goal of our study was to detect SNPs in Barnacle Goose by using a reference genome from a different bird species, the Mallard (Huang *et al. in prep*), knowing that geese and ducks diverged approximately 30 million years ago [22]. The method presented can be of practical benefit for SNP detecting in other species.

## Methods

### Sample Collection and Preparation

The SNP discovery panel consisted of ethanol preserved whole blood samples from 16 individuals from Spitsbergen, The Netherlands and Russia (Table 1) (The Dutch blood samples were collected under permit 4772A DEC (Animal Experimental Committee) University of Groningen and Ontheffing Flora- en faunawet: FF/75A/2007/032. Animal material was imported under permit Import ontheffing dierlijke bijproducten: TVWA/06/56935). We isolated DNA using the Gentra Systems Puregene DNA purification kit as described in [9]. We made two reduced representation libraries (RRLs) from a DNA pool of the discovery panel individuals with the restriction enzymes *Alu*I and *HaeIII*. The RRL size ranged from 100 to 150 bp. We pooled equal amounts of the two RRLs and submitted them for sequencing on the Illumina GAII (Illumina Inc., USA) using the Illumina Sample Preparation protocols [23]. Paired-end sequencing was performed for 101 cycles. For validation by genotyping we used the same individuals as those used for the discovery panel. In addition, we collected 26 samples from barnacle geese, originating from Greenland and the wintering population in the Netherlands, from museum samples from the Zoological Museum Amsterdam. We obtained the samples from pieces of flesh from the foot and we isolated DNA in the same way as described above. Different from the blood samples, we repeated the Proteinase-K treatment several times because the tissue was very tough. As the tissue did not dissolve enough to allow Proteinase-K to work effectively, we further destructed the tissue by holding the tubes containing the samples in liquid nitrogen until they were completely frozen. Then, we took them out until they were completely thawed, and repeated this five times. Thereafter we had another few steps with Proteinase-K until the tissue was dissolved. We evaluated the DNA fragments of the museum samples for quality on agarose gels and measured quantity and purity on a Nanodrop ND-1000. We diluted all samples (16 from discovery panel and 10 from museum) to 50 ng/ul for genotyping.

**Table 1:** Numbers of used individuals per location for the SNP discovery panel.

Population	Coordinates (lat; long)	Number of individuals
Spitsbergen – Nordenskioldkysten	77.8°; 13.6°	3
Spitsbergen - Ny-Ålesund	78.92°; 11.91°	4
Russia - Nova Zembla	71.4°; 54°	2
Russia – Kolguev	69.1°; 49.9°	2
Russia – Kanin	68°; 45°	2
The Netherlands - Krammersche Slikken	51.6°; 4.2°	3

### In silico SNP Mining

Quality filtering of raw reads was carried out by Perl scripts. Due to the use of the restriction enzymes *Alu*I and *HaeIII* all sequences should start with a cytosine (C). Sequences not starting with ‘C’ were therefore discarded from the dataset. We trimmed all reads beyond position 62, where the average phred quality score per base position [24] dropped below 17. We treated sequence reads occurring in at least two identical copies in this subset as reliable, making quality checks for these specific reads unnecessary [20]. We discarded any singleton sequence containing a nucleotide with a quality score of less than 15 as unreliable. Based on the raw sequence coverage of our RRLs (38×) we also excluded reads suspected to stem from repetitive regions by applying a fourfold overabundance threshold [20].

We aligned the resulting (quality filtered) reads to the reference genome with default parameters in MAQ [25]. Due to the lack of a sequenced goose genome we used mallard genome scaffolds (Huang *et al.* in prep) as a reference. The divergence time between mallard and the genus *Branta* is 28.1 Mya [22]. We considered only unambiguously mapped reads for SNP calling. Furthermore we filtered the candidate SNPs as predicted by MAQ according to the following criteria: minimal map quality per read: 10; minimal map quality of the best mapping read on a SNP position: 60; maximum read depth at the SNP position: four times the actual coverage after quality filtering; minimum consensus quality: 30. In addition we discarded SNP sites with a minor allele count of 1 or 2 as potential sequencing errors [20], [21].

From the aligned Barnacle Goose reads we made a consensus file in MAQ to retrieve 50 bp flanking sequences of the SNPs on both sides. Whenever there were no flanking sequences available from the Barnacle Goose consensus, we used the flanking sequences obtained from the Mallard genome, resulting in a *chimeric* flanking sequence from both Mallard and Barnacle Goose. We retrieved all flanking sequences using ad hoc *R*-scripts [26]. We used the amount of bases that originated from the Barnacle Goose consensus as a selection criterion for the 384 SNP genotyping set, because the genetic distance between Mallard and Barnacle Goose may be a cause of failure during genotyping, and hence we chose the SNPs with predominantly Barnacle Goose flanking sequences.

We mapped the detected SNPs against the Chicken genome *Gallus gallus* [27] (WASHUC2) using Blastn [28] with default settings. We used the Chicken genome, because it is the closest related species of which a physical genome map is available (divergence time is 81.2 Mya [22]), thereby allowing us to predict the likely chromosomal position of the SNPs. Because of the high degree of conserved synteny between birds, this allows us to select evenly spaced SNPs in the goose, even in the absence of a goose genome sequence. As final selection criteria we used 1) the distribution of SNPs across the chicken genome to minimize physical linkage and dependence among the selected SNPs and 2) an Illumina assay design score of >0.8. Because of a higher recombination rate on the micro-chromosomes in birds we used a smaller SNP spacing for the micro-chromosomes (Table 2). Because we used a small number of individuals for the SNP detection we analyzed the frequency distribution of the minor allele frequencies (MAF) to assess the ascertainment bias. Additionally we calculated the transition/transversion (TS/TV) ratios for the detected and selected SNPs.

**Table 2:** Minimum distances between SNPs on the Chicken genome and the number of SNPs used in the 384 genotyping set per chromosome.

Chromosome	Distance (kb)	Number of SNPs
1	200	57
2	200	56
3	200	34
4	200	31
5	200	28
6	150	9
7	150	16
8	150	18
9	150	13
10	150	10
11	100	7
12	100	16
13	100	13
14	100	5
15	100	5
17	100	7
18	100	3
19	100	9
20	100	11
21	100	5
22	100	1
23	100	3
24	100	9
26	100	2
27	100	1
28	100	2
Z	200	12

### Validation

For validation by genotyping we used all 16 individuals of the discovery panel, which were genotyped for 384 SNPs with the Illumina Golden Gate® genotyping assay on an Illumina® BeadXpress with VeraCode™ technology as described in Kraus *et al*. [9]. In contrast to the pre-validation, we based assay primers for each SNP on the *chimeric* flanking sequences, i.e., as many as possible bases in the flank sequences originated from the Barnacle Goose and where not enough were available Mallard sequence was used. We performed the allele calling (clustering) with the program Genome Studio (Illumina). We calculated the observed MAF for each SNP with CoAncestry [29] by taking the frequency of the least frequent allele and averaged that over all loci to obtain average MAF. In addition to the individuals of the discovery panel, we genotyped the five best museum samples originating from Greenland and the five best samples from wintering barnacle geese in The Netherlands (Table 3). We defined samples as ‘best’ that had both sufficient amounts of DNA and were of sufficient fragment lengths (sample codes: ZMA5090, ZMA5091, ZMA16572, ZMA17154, ZMA21106, ZMA27175, ZMA28449, ZMA28451, ZMA28453 and ZMA29205).

**Table 3:** Details of Museum samples.

Population	Coordinates (lat; long)	Year of sampling
Greenland	70.52°; −22.30°	1973
Greenland	70.26°; −22.37°	1974
Greenland	70.34°; −22.36°	1974
Greenland	70.57°; −22.30°	1974
Greenland	70.48°; −22.27°	1975
The Netherlands	51.44°; 04.02°	1947
The Netherlands	51.44°; 04.02°	1947
The Netherlands	51.42°; 04.28°	1962
The Netherlands	53.24°; 06.08°	1963
The Netherlands	52.35°; 05.53°	1929

## Results

**Figure 1:**
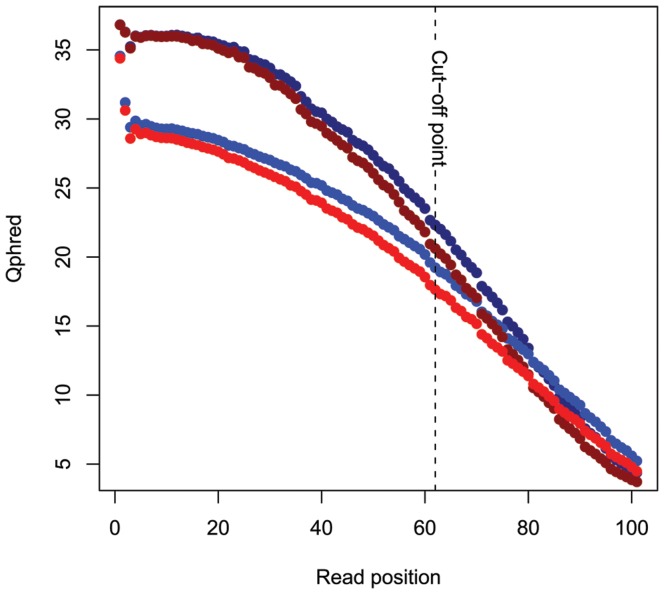
Phred quality scores per position. Average phred scaled quality scores of two paired-end lanes of 101 bp. The dotted line indicates the cut-off point for further analysis and shows that the minimum average quality score on position 62 is 17 (error prob.: 1/50.12). The different colours indicate the different lanes. One paired-end lane is plotted in dark blue and light blue (different colours for the different read directions) and the other in dark green and light green.

**Figure 2:**
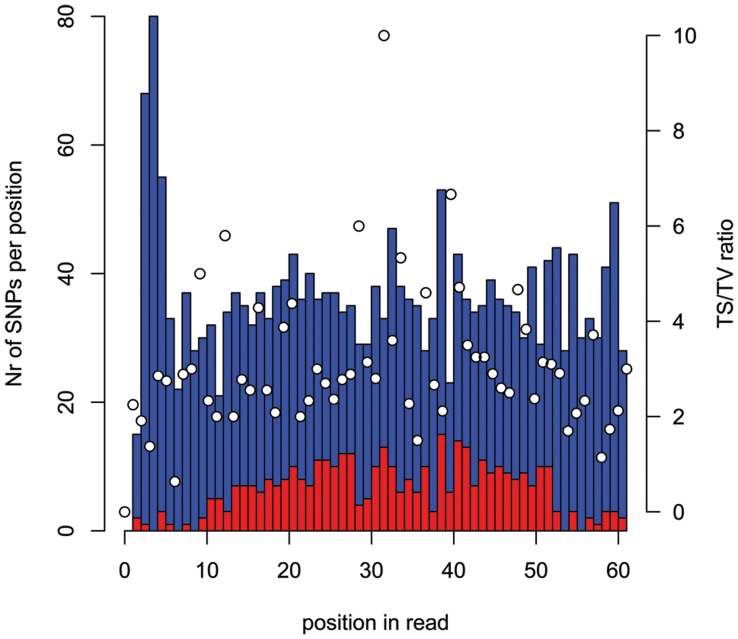
Detected and selected SNP per position. The number of detected (blue) and selected (red) SNPs per read position (scale on the left y axis). The white dots indicate the TS/TV ratio for the detected SNPs per position (scale on the right y axis).

**Figure 3:**
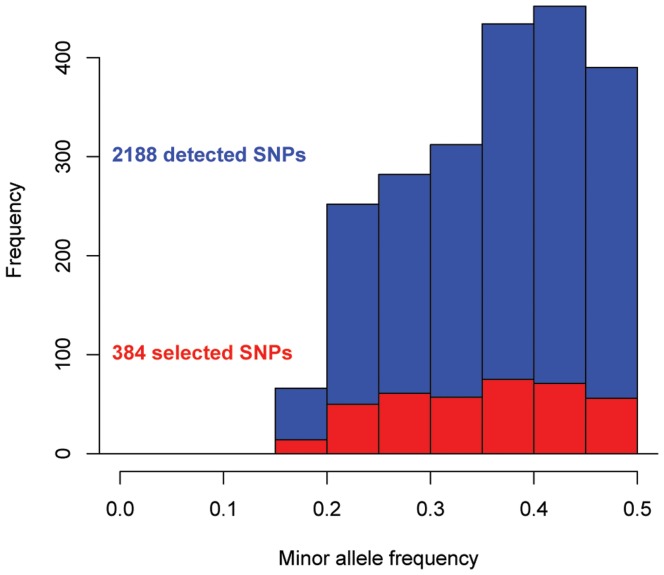
Minor allele frequencies. Minor allele frequencies (MAF) of detected (blue) and selected (red) SNPs. Mean MAF of detected SNPs was 0.37, mean MAF of selected SNPs was 0.36. The inserted box plots show the median MAF of both the detected (blue) and the selected (red) SNPs.

**Figure 4:**
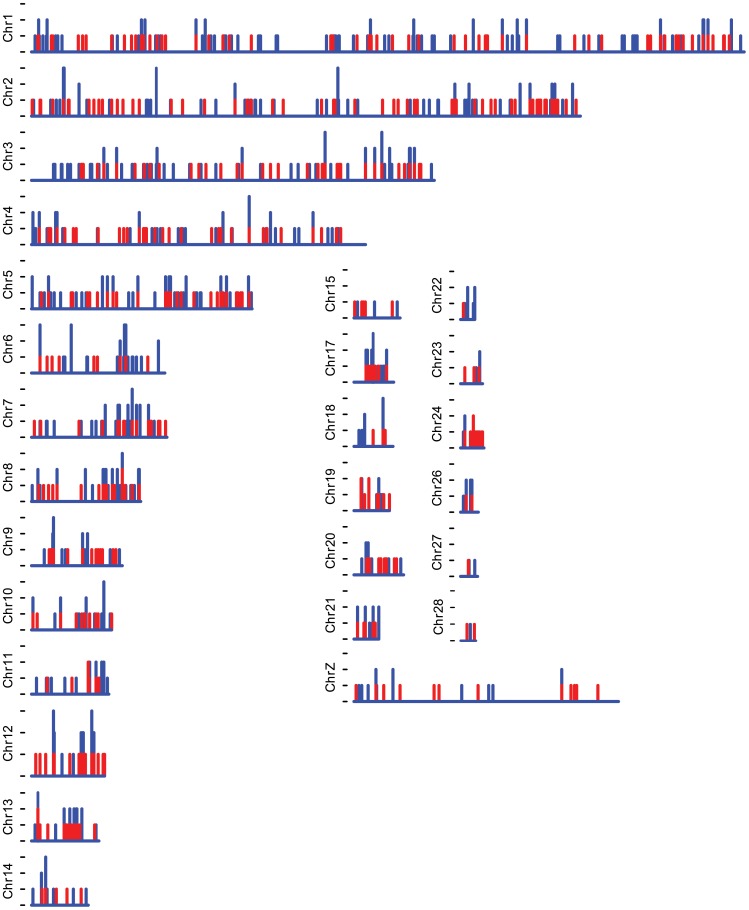
Map of detected and selected SNPs over the chicken genome. Per chicken chromosome the number of detected SNPs (blue) and selected SNPs (red) per 200 kb bin is shown. Because the bin size is 200 kb, and the minimum distance between selected SNPs is less than 200 kb for the smaller chromosomes, two SNPs per bin occurred in chr 8, chr 11, chr 13, chr 19 and chr24. The y-axis shows the number of SNPs per bin with one per tick.

We obtained 25.8 million reads of 101 bp length (2.6 billion nucleotides) using paired-end sequencing on two lanes of an Illumina GAII, representing approximately 5% of the genome with an estimated sequence depth of 38× (Figure 1). The raw sequencing data has been deposited in the NCBI sequence read archive (SRA) under accession number SRA029107. The number of 62 bp reads that passed the quality filters was 11 million (683.4 million nucleotides), providing a sequencing depth of 9.9×. We based these calculations on 5% coverage, which was an over-estimation because of the gaps in the middle of the larger RRL-fragments due to read trimming. The actual percentage of the Mallard genome that we could align our reads with was 1.48% (16.4 Mb of bases in Goose consensusfile/1.105 Gb in Mallard genome). Of these 11 million sequences 1.77 million (16.1%) aligned to the Mallard genome (Huang *et al.* in prep) which resulted in 363,014 candidate SNPs (mostly between Mallard and Barnacle Goose) as inferred by MAQ, of which 2188 SNPs (0.6%) passed all quality criteria. These SNPs have been deposited in the NCBI dbSNP database under accession numbers ss295471227 through ss295473414 for internal SNP identifiers Ble_1 - Ble_2188. We obtained 377 SNPs with at least 30 bp of goose consensus sequences on both sides of the SNP, 647 with 20–29 bp on both sides and 586 with 10–19 bp on both sides. The amount of SNPs detected per position on the reads was uniformly distributed (*t* = 1.06, *d.f*. = 2187, *p* = 0.29, Figure 2). The predicted mean minor allele frequency (MAF) of the 2188 SNPs, as inferred from sequencing the discovery panel RRLs, was 0.37 (figure 3), indicative of ascertainment bias as has been shown for this sort of SNP detection [8], [30]. A total of 923 SNPs could be mapped to unique locations distributed evenly over the chicken genome (figure 4), where we did not limit ourselves to evenly distributed locations. The TS/TV ratio of all SNPs was 2.7. The selection of the 384 SNPs for genotyping did not result in a bias with respect to selected SNPs per position (figure 2, in red, mean position selected) and predicted minor allele frequency (figure 3, in red).

### Validation

The validation by genotyping, for which we used all 16 discovery panel individuals and the ten museum samples, showed that 374 (97%) of the 384 assayed SNPs gave reliable genotypes in the assay and 358 (96% of the 374) were polymorphic. The quality of the historical samples was initially thought to be insufficient for SNP detection due to high fragmentation of the DNA. Of the initial 26 historical samples we used ten samples, despite the agarose gel showing high degradation, for genotyping and our worst performing sample of these then still had a success rate of 80% for the 374 SNPs. The lowest call rate among our discovery individuals was 91%. The heterozygosity of the genotyped discovery individuals was 0.34 and the measured mean observed MAF was 0.29. There was no effect of sequencing position in the read or origin of flanking sequence (proportion stemming from Barnacle Goose) on the technical failure of SNPs (position: *χ^2^* = 59.1, *d.f.* = 63, *p* = 0.62; flanking origin: *χ^2^* = 4.16, *d.f.* = 3, *p* = 0.25).

## Discussion

The genome wide SNP development in this study is, to our knowledge, the first for a fully migratory bird and the first in which a reference genome from another species was used. Previous genetic marker sets for goose species only included a small number of microsatellites [5], [6], [31]–[33], which have considerably less statistical power than the large number of SNPs we identified [7].

Despite using a relatively small discovery panel and limited read depth (<10×), our distribution of MAF shows that also relatively low-frequency SNPs could be detected, which may be especially useful for discriminating populations. The TS/TV of 2.7 for the detected SNPs is comparable to the TS/TV ratios described in other studies [9], [21]. This high TS/TV ratio in general is a good measure for a low frequency of false positives in the SNP discovery analysis, which is also confirmed by our high SNP validation rate of 97%.

The museum samples that we genotyped performed with a minimum success rate of 80%. This provides opportunities for using relatively old highly degraded museum samples for SNP genotyping with the Illumina Golden Gate® genotyping assay, provided that sufficient quantities of DNA are available. Caution should be taken however, as we selected those samples that we expected to have the largest chance of successful genotyping. We did not genotype all museum samples as it was not the main priority of our genotyping assays. Studies using only such museum samples should take potential loss of samples into account in the design. Still, earlier SNP genotyping of highly degraded DNA samples was tedious and only possible on low automation and throughput [34].

Approximately 16% of our reads (that passed the quality filters) aligned to the Mallard genome. Because we obtained our SNPs from these reads, it is not surprising that also the nearby sequences from Mallard provided good flanking sequences for genotyping, because we apparently have a bias for SNPs in the better conserved regions of the genome. This extreme sequence conservation between the genera *Anas* and *Branta*, both belonging to the family *Anatidae*, corroborates earlier findings of highly conservative genome evolution in birds [35], a fact that has previously been exploited for targeted gene marker development in highly conserved genomic regions in birds [36]. Especially in waterfowl (ducks, geese and swans) there seems to be an elevated potential to share polymorphisms between species [37].

Our results show that our method, in which we used the genome of the Mallard, provides excellently performing SNPs. We show that there is no effect on the performance of the SNP assay of the origin of flanking sequences in the assay design between these two species. Both SNPs with a high percentage of flanking sequences of Barnacle Goose and SNPs with a high percentage of flanking sequences of Mallard worked very well, and we observed no difference in their overall performance during genotyping. To our knowledge this is the first study in which *chimeric* flanking sequences are used successfully. We show that an RRL can be used to obtain SNPs and flanking sequences by aligning to a related species of the focal species in birds.

With the current developments, sequencing costs are rapidly decreasing, which will make the use of RRLs redundant. However, in this study with an RRL approach we are able to demonstrate that our method could work equally well when scaled up to whole genome sequencing of a discovery panel of individuals using a reference genome of a related (bird) species. This makes the complicated steps of a *de novo* assembly for the focal species [20], [21] unnecessary for SNP detection aimed at medium sized SNP sets of a few hundred to a few thousand SNPs. Given our RRL size of 5% of the Barnacle Goose genome, and our 2188 detected SNPs therein, scaling up to a whole genome approach is expected to yield more than 43.000 SNPs.

This genome wide SNP development of the Barnacle Goose provides us with a tool to study the genetic effects of population, and possibly migration, changes within a species that is renowned for its flexibility in migration [13]–[15]. The successful use of *chimeric* flanking sequences for genotyping our SNPs is in line with earlier findings and expectations for bird genome evolutionary patterns. Additionally, our study shows that the detection of thousands of assayable SNPs is now within reach for many more species than there is detailed genomic information for.
